# Perceived barriers and enablers influencing health extension workers toward home-based hypertension screening in rural northwest Ethiopia: interpretive descriptive study

**DOI:** 10.1186/s12913-022-08523-z

**Published:** 2022-09-13

**Authors:** Destaw Fetene Teshome, Shitaye Alemu Balcha, Tadesse Awoke Ayele, Asmamaw Atnafu, Mekonnen Sisay, Marye Getnet Asfaw, Getnet Mitike, Kassahun Alemu Gelaye

**Affiliations:** 1 Department of Epidemiology and Biostatistics, Institute of Public Health, College of Medicine and Health Sciences, University of Gondar, Gondar, Ethiopia; 2 Department of Internal Medicine, School of Medicine, College of Medicine and Health Sciences, University of Gondar, Gondar, Ethiopia; 3 Department of Health Systems and Policy, Institute of Public Health, College of Medicine and Health Sciences, University of Gondar, Gondar, Ethiopia; 4 Department of Human Nutrition, Institute of Public Health, College of Medicine and Health Sciences, University of Gondar, Gondar, Ethiopia; 5 Department of Emergency Nursing, School of Nursing, College of Medicine and Health Sciences, University of Gondar, Gondar, Ethiopia; 6 International Institute for Primary Health Care-Ethiopia, Addis Ababa, Ethiopia

**Keywords:** Hypertension screening, Health extension workers, Barriers, Enablers, Ethiopia

## Abstract

**Background:**

Hypertension, a major but modifiable risk factor for cardiovascular diseases, is a global health problem including Ethiopia. In a limited infrastructure task sharing of hypertension screening for community health workers is a feasible strategy to improve hypertension management. Recent finding have shown that trained health extension workers (HEWs) can identify high blood pressure, which was effective and feasible. Identifying barriers and enablers for home-based hypertension screening by HEWs is crucial for its implementation. This study aimed to explore barriers and enablers that influence health extension workers’ home-based hypertension screening in the community.

**Methods:**

The interpretive descriptive design was implemented. In-depth interviews were conducted during October, 2020. A total of 26 participants including HEWs, supervisors, and heads of district health office were purposively selected. They were asked to describe their perception toward home-based hypertension screening by the HEWs. The interviews were audio-recorded, transcribed verbatim into Amharic, and translated into English. The transcripts were coded and themes were identified. Thematic approach was used for data analysis.

**Results:**

The participants identified key perceived barriers and enablers of HEWs home-based hypertension screening. The most common barriers were a lack of hypertension training, blood pressure measuring devices, blood pressure guidelines and manuals, skilled HEWs, financial incentives, and poor community awareness of the disease. The most common enablers were support from community leaders, presence of functional development army and community trust for HEWs, presence of routine campaign on vaccination and community based health insurance, and an integrated health system.

**Conclusions:**

Our findings have implications for the HEWs' ongoing implementation of home-based hypertension screening. Successful implementation of this strategy requires scaling up of hypertension training programs for health extension workers and their supervisors, provision of standardized protocols, provision of adequate blood pressure measuring equipment, and regular supportive supervision.

## Background

Hypertension often called the “silent killer” is a long-term medical condition in which the blood pressure (BP) in the arteries is persistently high [1]. Hypertension, the most important modifiable risk factor for coronary heart disease, stroke and other cardiovascular diseases (CVDs), is an important public health problem all over the world [2]**.** In Ethiopia, as in other developing countries, hypertension is the major modifiable risk factor for CVD morbidity and mortality [3], accounting for more than half of all CVDs deaths [4]. For example, hypertension was the predominant risk factor for CVDs in studies conducted at Ayder Comprehensive Specialized Hospital (36.7%) [5], Jimma University medical center (40.2%) [6], Saint Paul Hospital Millennium Medical College (46.7%) [7], and Gondar University Specialized Hospital (62.3%) [4].

Nearly one out of every five Ethiopian rural adults suffers from hypertension [8]. A few pocket studies conducted in Humera (15.2%) [9], Arba Minch HDSS (17.5%) [10], Dabat district (18.5%) [11], and Dabat and Gondar district (25.3%) [12] show that hypertension is prevalent in rural areas of Ethiopia. These group of people are more at risk for hypertension complications than the urban populations because of poor awareness about hypertension either due to poor health literacy or low level of education[13], long distance to healthcare services [14], poor access to health information [15], poor access to health care [16, 17], and poor health-seeking behavior [18].

People with hypertension may go years without knowing they have the condition [19]. Long-term high blood pressure (HBP), however, is major risk factors for ischemic heart disease, strokes, peripheral vascular disease, chronic kidney disease, atrial fibrillation, and pulmonary embolism [20–24]. Early screening for HBP can help identify high-risk groups, resulting in timely treatment, BP reduction [25], and better BP control [26, 27]. However, large proportion of the population is unscreened and unaware of their condition [12, 28], resulting in less treatment and BP control [29]. For instance, a study conducted in Ethiopia showed that 77% of the population never had their BP measured [30] and 60% were unaware of their hypertension status [12, 28]. Other authors have found that when a sample is screened for hypertension, the prevalence is 3.5 times higher than what the subjects reported [31].

Ethiopia’s Federal Ministry of Health intends to increase the proportion of diagnosed hypertensive adults and making patients aware of their condition from 40 to 50% by 2022 and 60% by 2025 as part of its second health sector transformation plan (HSTP-II) [32]. However, a lack of access to care and shortage of trained health care providers may limit the country’s capabilities to meet those targets, necessitating strategies to make better use of available resources. To alleviate the burden of a shortage of highly skilled health professionals in areas where access to health services is limited, the WHO recommends a task-sharing strategy that promotes mid-level health workers in clinical tasks [33]. For instance, in low-resource settings, task sharing of hypertension screening from high-level health care providers to lower-level health care provider was found to be an effective strategy [34–36].

Health extension program is one of the most innovative community-based health program in Ethiopia [37]. Health extension workers has been found to be effective in managing a variety of health conditions in Ethiopia, including malaria, HIV, tuberculosis, maternal and child health problems [38]. A recent study also found that trained HEWs can correctly identify HBP in reliable and valid way [11]. Hence, for a strategy to be successful, evidence on barriers that will impede HEWs' home-based hypertension screening intervention and solutions that enable the program to be real, must be identified. We conducted an interpretive descriptive qualitative study to describe the perceived barriers and enablers that will influence HEWsˈ participation in home-based hypertension screening. This study generates evidence on effective, practical, and sustainable intervention programs for lowering BP, controlling hypertension, and preventing cardiovascular disease.

## Methods

### Study design and setting

The interpretive descriptive qualitative study was carried out in the rural areas of Gondar Zuria and Dabat districts of northwest Ethiopia. It was conducted in October 2020 to explore barriers and enablers that influence HEWs’ home-based hypertension screening. Gondar Zuria is one of the districts in Central Gondar Zone of northwest Ethiopia. In this district, 42,753 households were counted, resulting in an average of 4.48 people per household. Dabat is one of the districts, and it is located in the North Gondar Zone. In this district, a total of 31,111 households were counted, resulting in an average of 4.68 people per household [39]. The details are described elsewhere [11].

### Current role of health extension workers in Ethiopia

Health Extension Workers are the key drivers of the health extension program. Each health post has two to three HEWs assigned to it. The HEWs are responsible for identifying pregnant women within their catchment area, providing antenatal care, and connecting them with the formal health system if there is a high risk or complications. They are also responsible for following women during the postnatal period, when care for both mother and newborn is critical [40]. The details are described elsewhere [11].

### Study participants and sampling strategy

This study was conducted as part of the research project “improving hypertension management through task sharing with the Health Extension workers in rural districts of northwest Ethiopia”. This study included HEWs, supervisors of HEWs, and heads of districts health office. Purposive sampling was used to recruit study participants. Data saturation is a criterion for determining sample size in qualitative interview studies. It means that if interviews with new respondents do not yield new themes, the number of respondents is sufficient. Accordingly, 17 HEWs (one from each candidate kebele), 7 immediate supervisors, and 2 districts health office heads took part in the study. We used the consolidated criteria for reporting qualitative research (COREQ) with a 32-item checklist to report the findings [41].

### Study tool development and training of the research team

A semi-structured interview guide with open-ended questions was used to explore perceived barriers that can make HEWs’ home-based hypertension screening difficult to perform, enablers that can make the screening intervention more accessible in the study settings, and solutions to be made the strategy to be successful. A thorough review of relevant literatures, available tools, and consultation with content experts were conducted. The interview guide was prepared in English, reviewed by the experts for face validity, and then translated into the local language (Amharic). The researchers have more than 10 years of teaching and research experience. The research team was trained for three days to ensure that they understand the study’s objective, the research tool, and how to collect data from participants. They were also taught how to handle sensitive situations appropriately by using appropriate wording, supportive statements, and avoiding excessive probing. A pre-test was done in the study setting to ensure cultural and contextual appropriateness of the interview guide to the Ethiopian context.

### Data collection procedures

Three researchers (DFT, TYA, and MS) fluent in English and Amharic conducted individual face to face in-depth interviews in Amharic. They used a semi-structured interview guide for all the informants to describe their perception about the HEWs home-based hypertension screening intervention. This method enabled interviewers to customize questions to the needs of the various participants. Participants were asked probing questions during the interview to provide more detail on their responses. Data were collected until saturation was reached. Each in-depth interview lasted 20–31 min. All interviews were conducted privately at the participants' workplaces. All in-depth interviews were recorded using voice recorders. A research assistant also took notes on the proceedings of the session. Information exchange by telephone and close supervision by the principal investigator and supervisor were made on a daily basis.

### Data analysis

All audio recordings from in-depth interviews were transcribed verbatim into local language (Amharic) and translated into English by the individual who conducted the interviews. An inductive approach was used to analyze the data. The data were then analyzed in two steps. Initially, the primary investigator (DFT) and a co-investigator (MGA) independently read and reviewed the transcripts for accuracy. The researchers identified sections containing information about perceived barriers and enablers of HEWs’ home-based hypertension screening. Second, all of the selected sections of the interview transcripts were manually coded and thematically analyzed. To illustrate each important theme identified, quotations from the data were used. The codes and themes were also discussed and decided upon. To increase the credibility of our analysis, we used a pretested interview guide, experienced and trained data collectors (credible researchers), and the analyst triangulation method. Moreover, the credibility of the data was ensured using member checking during and after data collection to verify the information gathered and the interpretation of our findings. The data collection and analysis from the in-depth interviews followed the Standards for Reporting Qualitative Research (SRQR).

## Results

### Participants characteristics

This study included 17 HEWs, 7 HEWs’ supervisors, and two district health office heads. Participants were middle-aged, ranged from 24 to 38 years. All of the HEWs, as well as two of the supervisors, were female. The majority of the HEWs had more than 10 years of work experience, ranging from one to fifteen years (Table 1).

**Table 1:** Characteristics of participants (*n* = 26)

Characteristics	Number
Type of Health care worker	
Health Extension workers	17
Supervisors	7
Head of the district health office	2
Sex	
Male	7
Female	19
Age	
21–30	12
31–40	14
Work experience	
1–5	7
6–10	5
11–15	14

### Perceived barriers for home-based hypertension screening by HEWs

We identified four themes and seven subthemes of barriers to implementing a HEWs’ home-based hypertension screening intervention, including: low skilled HEWs; lack of training, equipment, guidelines, or manuals; inadequate human power; lack of financial incentives; lack of supportive supervision, and lack of community awareness of the disease (Table 2).

**Table 2:** Healthcare providers’ perspectives on barriers to task sharing in hypertension screening with HEWs by theme

Themes	Subthemes	
**Human resources**	Human power	Low skilled HEWs (R18, R23, R24, R25)
**Material resources**	Training	Lack of training (R01, R02, R03, R04, R05, R06, R07, R13, R14, R16, R19, R20, R25)
	Blood pressure measuring equipment	Lack of BP measuring equipments (R02, R03, R04, R05, R06, R08, R09, R11, R13, R14, R15, R16, R18, R20, R21, R22, R25, R26)
	Guidelines and manuals	Unavailability of manuals and guidelines to measure BP and classify BP readings(R08, R18, R20, R24)
	Financial incentives	Lack of financial incentives for the HEW and development army (R05, R08, R13, R18, R24, R25, R26)
**Health system related**	Supportive supervision	Lack of supportive supervision (R03, R04, R13, R14, R20)
**Community awareness**	Perception about hypertensive disease	Lack of community awareness of hypertension (R06, R18, R20, R21, R24, R26)

### Low skilled health extension workers

According to some interviewees, one of the barriers to implementing HEWs’ home-based hypertension screening is low skilled health extension workers. One of the participant described that *“One of the reasons we won’t be able to share this activity to the HEWs is that they lack the necessary knowledge and skills. That is, if she has a knowledge gap, she may not be able to check it confidently* (R23, HEW supervisor). Another participant also stated *“…as I told you before, there may be skill or knowledge gaps in terms of measuring BP and answering questions from the community.”* (R25, HEW supervisor). *One of the head of the district health office also said: “Not all HEWs are the same. Some of them have difficulty measuring BP”* (R24, Head of district health office).

### Lack of training regarding hypertension

The majority of the HEWs emphasized the importance of home-based hypertension screening by the HEWs, while focusing on the need of training. One HEW stated: *“I believe a lack of training is one of the barriers to implementing hypertension screening at the community level by the HEWs”* (R04, HEW). One of the HEWs’ immediate supervisors also mentioned the importance of home-based hypertension screening by the HEWs. *“Because our primary goal is to save the health of the community, I don't think it would be a problem if we did this together. However, HEWs must take the training seriously”* (R19, HEW supervisor).

### Lack of blood pressure measuring devices

Availability of BP measuring equipments are the most important prerequisites for home-based hypertension screening by HEWs. The majority of participants stated that BP measuring equipment such as sphygmomanometer and Stethoscopes are required to carry out this program. The majority of HEWs described the lack of BP measuring equipment in the health post is the main barrier to implement HEWs home-based hypertension screening intervention in the community. One HEW expressed her concerns about lack of BP measuring device in the health post by saying: *“If we have a functional BP measuring device in hand, there is nothing stopping us from measuring individuals. We can, of course, view training as a challenge. However, if we think about the affected community, even if the health extension workers are stressful, we may not be concerned too much about the workload”* (R02, HEW). Another HEW expressed her thoughts on the necessity of BP measuring device as follows: *“It would be great if we had a BP measuring device to save our people from the effects of HBP. We all have no problem doing such activities now that if everything is in place”* (R09, HEW). One more HEW emphasized the importance of blood measuring device and training, saying, *“The message I want to transfer to the Ministry of Health is that BP measuring devices must be met. We are willing to work if the BP apparatus is provided****”*** (R13, HEW). The following is how one of head of the district health office explained the need for BP measuring materials for the HEWs. *“They will need BP measurement equipments such as BP measuring device, a stethoscope, and materials that explain what BP means in order to measure it. These medical devices are required because there is a scarcity of materials and measuring instruments on the market. Registration books and manuals with BP definition and disease stages must also be prepared for HEWs”* (R18, Head of district health office). 

### Unavailability of guidelines or manuals to measure blood pressure

Most of the participants believe that lack of standard guidelines and manuals will make it difficult to implement home-based hypertension screening. One of the participant said that *“…I don't believe the service can be launched until the guidelines are available at all health post levels”* (R24, Head of district health office). The other participant also said that *“…Registration books and manuals with BP definition and disease stages must also be prepared for HEWs”*(R18, Head of district health office).

### Lack of financial incentives

Some participants perceived a lack of financial support to be one of the most significant barriers to implement home-based hypertension screening by HEWs. “In the past, there were non-governmental organizations that helped us. Nowadays we are in a lot of trouble after these non-governmental organizations diminished their support. We need an incentive at least once every 4 to 6 months to encourage HEWs. The development army are also exhausted at this point. A HEW supervisor working in one of the districts said that: *“When we asked the heads of the district health offices for incentives for the health development army, they began to calculate the budget, which came to hundreds of thousands of birr, and it was not possible to pay these people financially. In this regard, development teams are exhausted and out of work. If we are supported, it will be easier for us to do this work”* (R26, HEW). In contrast, another participant stated that if home-based hypertension screening is shared with HEWs, a lack of incentives will not be a major barrier. He stated that *“I don’t think lack of incentives appears to be an impediment to sharing these activities to the HEWs”* (R24, Head of district health office).

### Lack of supportive supervision

Some participants stated that supportive supervision is required in order to have a successful home-based hypertension screening by the HEWs. One participant stated the importance of supportive supervision of the HEWs *“First, HEWs must be trained in skill development, and then BP measuring instrument should be in place in all health posts. The other is that health centers should support HEWs. Just because a person is trained does not mean that he or she works”* (R14, HEW). One of the HEWs’ supervisors also mentioned the importance of supportive supervision for the task sharing strategy to be effective. *“Not only does the program need to be included, but also needs to be monitored. Giving an assignment to a person and ignoring him means helping a person sleep but not waking him up. As far as I know, the first and most important thing that we can do to prevent HBP is to get HEWs ready. This means that health workers from the federal level down to the kebele level should be ready to save the people* (R20, HEW supervisor). 

### Lack of community awareness of the disease

Some participants believed that poor community perception of the disease would impede the implementation home-based hypertension screening by HEWs. One participant said that: *“The challenges can be seen in many ways. When you come to the affected community, the first thing you notice is that people are ignoring their HBP and not following the instructions that their health care provider gives them*” (R18, Head of district health office). One of the HEWs’ supervisors agreed and stated, *“The major barrier is poor community perception of the disease. That is, if you tell them you have HBP and don’t eat this, don’t drink this, don’t do this, and do exercise, they will not understand easily”* (R26, HEW supervisor).

### Perceived enablers for HEWs’ home-based hypertension screening

Several enablers for engaging HEWs in home-based hypertension screening categorized in to two themes and six subthemes were identified in this study. Support from community leaders, the presence of various community development armies, community trust for HEWs, the presence of routine campaigns on vaccination and community based health insurance, an integrated health system, and proximity of the community to the health facility are all enabling factors (Table 3).

**Table 3:** Healthcare providers’ perspectives on enablers to task sharing in hypertension screening with HEWs by theme

Themes	Subthemes	
**Community support systems**	Community leaders and groups	Support of community leaders such as religious leader, elders, and kebele administrators (R01, R04, R7, R18)
	Presence of various functional development army	Presence of a 1 to 5 development teams (R04, R05, R10, R14, R18, R19, R22)
		Presence of women’s development group (R12)
	Community trust	Good community attitude towards the HEWs (R01, R03, R05, R11, R14, R15, R16, R17)
**Health system related**	Routine campaign and CBI	Presence of routine campaign (R06, R18, R22)
		Presence of community based health insurance (R07)
	Integrated health system	Presence of integrated system (R10, R11, R24, R25, R26)
	Health facility	Proximity of the kebele to the health post (R11, R13)

### Support from community leaders

One of the opportunities to perform home-based hypertension screening by HEWs were getting support from the community leaders. This was echoed as follows: *“The opportunities to implement home-based hypertension screening at the community level are very welcoming of the kebele leaders for the program and have a good help to work. The kebele leaders are enthusiastic about the program and eager to help HEWs in mobilizing community members”* (R01, HEW).

### Presence of various functional community development army

The presence of various development armies in each kebele, such as 1 to 5 development network and women’s organizations, is the main opportunity described by informants, which may facilitate home-based hypertension screening by HEWs: *“The good thing is that we can raise the issue of hypertensive disease at the church on Sundays and holydays in collaboration with kebele special organizations, namely the kebele administration and women's organization”* (R04, HEW). *“We can take the existence of a 1 to 5 development group as the ideal condition, and the community's attitude toward it as very good”,* said a 35-years-old HEW with a level 4 educational status (R05, HEW). This idea was also supported by the HEWs’ immediate supervisor, who stated, *“One of the best things to do in the community is to have a 1 to 5 organization and development team. It's a great opportunity to work with them”* (R22, HEW supervisor).

“*It is convenient to provide the service for the affected community because we have the support of women's development groups. This is one good opportunity*” (R12, HEW). One of the HEWs supervisor was also supported this idea: “*The best opportunities to do these activities are having women's organization at the kebele level. There are also committees from the education, agriculture, and health departments that may be of assistance to the HEWs. There are community-based routine activities that should be done every month with religious leaders, elders, teachers, and government officials”* (R18, Head of district health office).

### Community trust for the health extension workers

The trust of communities and individuals in HEWs was perceived as vital enabling factor in implementing home-based hypertension screening by HEWs. Nearly half of the HEWs believed that providing hypertension screening for the community increases their acceptance as the HEW. A 32 years old HEW said that: *“The people are very welcoming and have good attitude with us. When we taught them at church, they said “you measured the BP for some of the kebeles, why not for others”? I informed them that this one was required for research purpose.”*(R01, HEW).

One HEW also said that: *“HEWs are not far from the community. As we live with the community. We have a good opportunity to reach out people and provide services easily because we live in the community”* (R14, HEW). A 27 year old HEW with 9 years of experience described the community’s attitude toward them as one opportunity to implement home-based hypertension screening by the HEWs as follows: *“The service is free, and the community has a positive view for us. They see us as mothers, brothers, and fathers, and it ‘s a good opportunity to come as an additional package”* (R17, HEW).

### Presence of routine campaign on vaccination and community based health insurance

Some participants highlighted the presence of routine campaigns as opportunities to implement home-based hypertension screening by the HEWs. *“There are some remote areas that are inaccessible”,* said one HEW. *“However, we have a campaign that will run alongside, and we will be able to reach them during the campaign. Therefore, it is a good opportunity to work together with the campaign”* (R06, HEW).

A 30 year old participant with 6 years of work experience agreed with the previous HEW’s idea and stated that: *“…..Health extension workers operate 85% of their work from house to house, making it easier for them to go and measure it. This is an effective approach to measure BP”* (R18, HEWs supervisor). One of the participants perceived that the presence of community-based health insurance in the community as an opportunity to implement hypertension screening in the community with the help of HEWs. *“We go from home to home to making them pay for a community-based health insurance, which we usually find the elderly people and we can easily do it together”* (R07, HEW).

### Integrated health system

Some of the participants believed that the presence of an integrated health system was critical in implementing home-based hypertension screening by HEWs. *“There are mothers who came for family planning service and it would be a good opportunity to measure them”*, said a 35-year-old HEW (R10, HEW). A 38-year-old supervisor agreed with the previous idea, saying, *“It is good to include as one package in the health extension program. If the service is started at the health post level, there is nothing wrong with our organization because it is being recognized. We have a complete HEWs that we call convenient conditions. Everything is complete”* (R26, HEW supervisor).

### The community’s proximity to the health post

The HEWs also felt the proximity of the kebele to the health facility as one of the opportunity to implement this task sharing strategy of hypertension screening with the HEWs. *“The proximity of the health post and the presence of a trained professional provide opportunities for HEWs to share hypertension screening"* (R13, HEW).

## Discussion

Our findings showed that HEWs are eager to do home-based hypertension screening and disease prevention. They did, however, explored various barriers that will impede their contribution to hypertension screening in their community. Participants expressed their concerns how to place this task-sharing strategy into action and keep it going. One of the potential barriers to implementing home-based hypertension screening intervention by HEWs is their lack of knowledge and skill about hypertension. Our finding is consistent with those from Nigeria [42] where community health workers expressed a need for noncommunicable diseases prevention and control staff capacity building. This could be because a health care worker with a lack of knowledge is unable to correctly measure BP.

Effective training of community health workers is critical for obtaining the knowledge and skill set required for hypertension screening and management performance [43]. However, in this study, HEWs had never been trained in hypertension. Participants in this study emphasized the importance of HEWs training for the successful implementation of HEWs’ home-based hypertension screening, which is consistent with research from Kenya [44], Ghana [45], Nepal [46, 47], Bangladesh [48], China [49], and India [50]. A systematic review conducted in low and middle-income countries also found that non-physician health care workers training, as well as the provision of algorithms and screening protocols, are crucial for implementing task sharing strategy [51]. This indicates the need for in-service training on hypertensive disease. The routine provision of refresher trainings is also important in reinforcing and updating skills and knowledge of the HEWs.

Lacks of BP measuring devices are the most important barriers cited by most of the participants in this study. Though the HEWs have the necessary knowledge and skills to provide home-based hypertension screening to their communities, they are often challenged by the context in which they work. Consistent with study conducted in Nigeria [42] and Bangladesh [48] lack of necessary BP measuring equipments for hypertension screening are considered to be a significant work related challenge to implement home-based hypertension screening expressed by most HEWs.

Some participants believed that unavailability of hypertension manuals and guidelines would be a barrier to implement home-based hypertension screening by the HEWs. Our findings are consistent with those of studies that looked at task shifting/sharing strategies for hypertension management. Studies in Nigeria [52], Nepal [46], and a systematic review in LMIC [51] revealed that inadequate use of guidelines by healthcare professionals impedes hypertension management. These supplies are very helpful to categorize individuals having hypertension or not and to take immediate action. This implies that the HEWs need to have the necessary equipment, guidelines, and manuals at each health post to make the strategy effective and sustainable.

Maintaining the motivation of CHWs to consistently conduct their responsibilities is a challenging, but crucial element in CHW programs. In this study, participants perceived that lack of financial support may influence the initiation and sustainability of this task sharing strategy for hypertension screening with the HEWs. This is consistent with the study conducted on Nurse-led task shifting in Ghana [45]. In study of a community-based health extension program in Ethiopia, researchers concluded that incentives and recognition of contributions could help to improve motivation and retention of HEWs [53]. Thoughtful consideration should be given to incentive structures as part of a strategy to retain trained CHWs.

In this study one of the most important perceived barriers to implement home-based hypertension screening by HEWs identified by most of the study participants was lack of community awareness about hypertension. This finding is consistent with studies conducted in Nigeria [42] and Bangladesh [48], where patients’ lack of awareness on noncommunicable diseases risk factors and symptoms were key challenges for hypertension screening at the community level.

Health extension workers commonly quote a lack of supervision and support from the health system as a barrier to implement home-based hypertension screening intervention by HEWs. This is consistent with study done in South Africa [54], Bangladesh [48], and systematic review in China [49] where lack of support by the government was one of the barriers perceived by the participants that may hinder the implementation of task sharing strategy of hypertension screening.

Despite these barriers, participants provided an ample of enabling factors that may help HEWs’ home-based hypertension screening intervention effective. Participants perceived that community leaders support, the presence of functional development army, community trust in them, the presence of routine campaign, and integrated health system as enablers that could help the task sharing intervention effective and sustainable.

The study revealed that positive community response to the program is a potential enabling factor that will facilitate HEWs’ home-based hypertension screening. It was consistent with a study in Bangladesh [48] where community support systems was one of the facilitating factors to engage community health workers in noncommunicable disease prevention and control.

Most of the participants perceived that community trust on the HEWs were one of the most enabling factors to engage HEWs in home-based hypertension screening. This finding was consistent with studies conducted in Kenya [44], South Africa [54], and China [49] where community trust on the community health workers served as a facilitator for the implementation of this strategy.

### Study strengths and limitations

This is the first study of its kind in Ethiopia to shed light on the task sharing strategy of HEWs for hypertension screening. The study explored the perceived barriers and enablers of home-based hypertension screening by HEWs in rural communities of Northwest Ethiopia. The findings of this study will inform the development of national policies and guidelines for involving HEWs in hypertensive disease prevention and control. However, this study did not include the perspectives of services recipients.

## Conclusion

We found that having low skilled HEWs, lack of training regarding hypertension, lack of BP measuring devices, guidelines and manuals, lack of financial incentives, lack of supportive supervision, and lack of community awareness of the disease may make it difficult for HEWs to implement home-based hypertension screening. Obtaining support from community leaders, the presence of functional development army and community trust for HEWs, the presence of routine vaccination campaign and community based health insurance, and an integrated health system are all opportunities for HEWs to implement home-based hypertension screening. This research found that hypertension measurement by HEWs is a feasible task sharing strategy. Successful implementation of this strategy requires: scaling up hypertension training programs for HEWs and supervisors, provision of standardized protocols, provision of adequate BP measuring equipment, and regular supportive supervision. With such systems in place there are substantial opportunities for major improvements in healthcare quality and outcomes for hypertension management in Ethiopia.

## Data Availability

This manuscript includes all data generated or analyzed during this study. We do not intend to share the data because it is a qualitative study with the raw data contains participant identifications, including their names. However data can be made available upon reasonable request from Destaw Fetene Teshome.

## Abbreviations

BP: Blood pressure
CVD: Cardiovascular Diseases
HBP: High Blood Pressure
HEWs: Health Extension Workers
LMIC: Lowe and Middle-Income Countries

## References

[1] Guwatudde D, Nankya-Mutyoba J, Kalyesubula R, Laurence C, Adebamowo C, Ajayi I (2015). The burden of hypertension in sub-Saharan Africa: a four-country cross sectional study. BMC Public Health.

[2] Drozdz D, Kawecka-Jaszcz K (2014). Cardiovascular changes during chronic hypertensive states. Pediatr Nephrol.

[3] Dagnaw W, Yadeta D, Feleke Y, Ethiopian KT, Guideline N, on Major NCDs,  (2016). Guidelines on Clinical and Programmatic Management of Major Non Communicable Diseases. Addis Ababa: FDREMOH.

[4] Tefera YG, Abegaz TM, Abebe TB, Mekuria AB (2017). The changing trend of cardiovascular disease and its clinical characteristics in Ethiopia: hospital-based observational study. Vasc Health Risk Manag.

[5] Bahrey D, Mariye T, Gebremedh G, Kassa A, Girmay A. Magnitude of Cardiovas-cular Risk Factors among Adult Hypertensive Patients Attending in Ayder Comprehensive Specialized Hospital, Ethiopia, 2018. International Archives of Cardiovascular Diseases. 2019;3:016.

[6] Ayele H, Banbeta A, Negash A. Cardiovascular Disease Risk Factors in Hypertensive Patients: A Case Study of Jimma University Medical Center. Health Services Research Managerial Epidemiology. 2022;9:23333928221078600. 10.1177/23333928221078601 PMC8848038 35187200

[7] Mengistu MD, Benti H. Assessment of magnitude and spectrum of cardiovascular disease admissions and outcomes in Saint Paul Hospital Millennium Medical College, Addis Ababa: A retrospective study. medRxiv. 2022. 10.1371/journal.pone.0267527 PMC10045542 36508450

[8] Tiruneh SA, Bukayaw YA, Yigizaw ST, Angaw DA (2020). Prevalence of hypertension and its determinants in Ethiopia: A systematic review and meta-analysis. PLoS ONE.

[9] Mengistu MD (2014). Pattern of blood pressure distribution and prevalence of hypertension and prehypertension among adults in Northern Ethiopia: disclosing the hidden burden. BMC Cardiovasc Disord.

[10] Chuka A, Gutema BT, Ayele G, Megersa ND, Melketsedik ZA, Zewdie TH (2014). Prevalence of hypertension and associated factors among adult residents in Arba Minch Health and Demographic Surveillance Site, Southern Ethiopia. PLoS One.

[11] Teshome DF, Balcha SA, Ayele TA, Atnafu A, Sisay M, Asfaw MG (2022). Trained health extension workers correctly identify high blood pressure in rural districts of northwest Ethiopia: a diagnostic accuracy study. BMC Health Serv Res.

[12] Abebe SM, Berhane Y, Worku A, Getachew A (2015). Prevalence and associated factors of hypertension: a crossectional community based study in Northwest Ethiopia. PLoS ONE.

[13] Islam F, Bhuiyan A, Chakrabarti R, Rahman MA, Kanagasingam Y, Hiller JE (2016). Undiagnosed hypertension in a rural district in Bangladesh: The Bangladesh Population-based Diabetes and Eye Study (BPDES). J Hum Hypertens.

[14] Agyemang C, Bruijnzeels MA, Owusu-Dabo E (2006). Factors associated with hypertension awareness, treatment, and control in Ghana, West Africa. J Hum Hypertens.

[15] Lloyd-Sherlock P, Ebrahim S, Grosskurth H. Is hypertension the new HIV epidemic? : Oxford University Press; 2014. 10.1093/ije/dyu019 PMC3937981 24491955

[16] Carey RM, Muntner P, Bosworth HB, Whelton PK (2018). Prevention and Control of Hypertension: JACC Health Promotion Series. J Am Coll Cardiol.

[17] Ababa A. Adressing the Impact of Noncommunicable Diseases and Injuries in Ethiopia. 2018.

[18] Azu CN, Onyeonoro U, Ogah OJJotACoC. PREDICTORS OF CARE-SEEKING BEHAVIOR AND INFLUENCE ON UNDIAGNOSED HYPERTENSION IN SOUTHEASTERN NIGERIANS. 2019;73(9 Supplement 1):1857.

[19] Mishra N, Chowdary K, Mishra G. HIGH BLOOD PRESSURE–“A SILENT KILLER”: IT’S PREVENTION AND THERAPY.

[20] Singer  D, Kite  AJEJoV, Surgery  E (2008). Management of hypertension in peripheral arterial disease: does the choice of drugs matter?. Eur J Vasc Endovasc Surg.

[21] Lau DH, Nattel S, Kalman JM, Sanders PJC (2017). Modifiable risk factors and atrial fibrillation. Circulation.

[22] Gaddam KK, Verma A, Thompson M, Amin R, Ventura  HJMCoNA (2009). Hypertension and cardiac failure in its various forms. Med Clin North Am.

[23] Reisin E, Jack  AVJMCoNA (2009). Obesity and hypertension: mechanisms, cardio-renal consequences, and therapeutic approaches. Med Clin North Am.

[24] Stroke and hypertension 29 may 2017 [Available from: https://www.world-heart-federation.org/resources/stroke-and-hypertension/.

[25] Kim Y, Radoias V (2018). Screening, diagnosis, and long-term health outcomes in developing countries—The case of hypertension. PLoS ONE.

[26] Schmidt B-M, Durao S, Toews I, Bavuma CM, Hohlfeld A, Nury E (2020). Screening strategies for hypertension. Cochrane Database Syst Rev.

[27] Krist AH, Davidson KW, Mangione CM, Cabana M, Caughey AB, Davis EM (2021). Screening for hypertension in adults: US Preventive Services Task Force reaffirmation recommendation statement. JAMA.

[28] Worku K. Addressing the impact of Noncommunicable Diseases and Injuries in Ethiopia: Findings and recommendations from the Noncommunicable Diseases and Injuries (NCDI) Commision of Ethiopia: A Collaboration with the Global Lancet Commission on Reframing NCDIs for the Poorest Billion Addis Ababa: Federal Democratic Republic of Ethiopia Minstry of Health; November 2018 [Available from: https://static1.squarespace.com/static/55d4de6de4b011a1673a40a6/t/5bfc17e24fa51a471a8399d9/1543247843790/Ethiopia+NCDI+Commission_Full+Report_Nov+2018.pdf.

[29] Ethiopia sets to improve hypertension prevention and control at primary health care level Ethiopia: World Health Organization; 2019 [Available from: https://www.afro.who.int/fr/node/11607#:~:text=Hypertension%20is%20one%20of%20the,getting%20appropriate%20treatment%20and%20care.

[30] Bekele A, Gelibo T, Amenu K, Getachew T, Defar A, Teklie H (2017). The hidden magnitude of raised blood pressure and elevated blood glucose in Ethiopia: A call for initiating community based NCDs risk factors screening program. Ethiop J Health Dev.

[31] Prevett M. Chronic non-communicable diseases in Ethiopia-a hidden burden. Ethiopian journal of health sciences. 2012;22(Spec Iss):1. PMC3542741 23319834

[32] Health Sector Transformation Plan II: HSTP II 2020/21–2024/25 (2013 EFY - 2017 EFY): Minstry of Health-Ethiopia; February 2021 [Available from: https://www.moh.gov.et/ejcc/sites/default/files/2021-05/HSTP-II.pdf

[33] Chan M. Task shifting: rational redistribution of tasks among health workforce teams: global recommendations and guidelines: World Health Organization, PEPFAR & UNAIDS; 2007 [Available from: https://apps.who.int/iris/handle/10665/43821.

[34] Deo S, Singh P (2021). Community health worker-led, technology-enabled private sector intervention for diabetes and hypertension management among urban poor: a retrospective cohort study from large Indian metropolitan city. BMJ Open.

[35] Gaziano TA, Abrahams-Gessel S, Denman CA, Montano CM, Khanam M, Puoane T (2015). An assessment of community health workers' ability to screen for cardiovascular disease risk with a simple, non-invasive risk assessment instrument in Bangladesh, Guatemala, Mexico, and South Africa: an observational study. Lancet Glob Health.

[36] James S, Sewpaul R, Reddy P, Madela S, Madela S (2020). Early detection, care and control of hypertension and diabetes in South Africa: A community-based approach. Afr J Prim Health Care Fam Med.

[37] Banteyerga H (2011). Ethiopia's health extension program: improving health through community involvement. MEDICC Rev.

[38] Zulliger R. Ethiopian Community Health Worker Programs [Available from: https://www.chwcentral.org/blog/ethiopian-community-health-worker-programs.

[39] Population and Housing Census of Ethiopia. Addis Ababa, Ethiopia: Central Statistical Agency; 2007.

[40] Caglia J, Kearns A, Langer A. Health extension workers in Ethiopia: Delivering community-based antenatal and postnatal care. Boston; 2014.

[41] Tong A, Sainsbury P, Craig J (2007). Consolidated criteria for reporting qualitative research (COREQ): a 32-item checklist for interviews and focus groups. Int J Qual Health Care.

[42] Aifah A, Onakomaiya D, Iwelunmor J, Oladele D, Gbajabiamila T, Obiezu-Umeh C (2020). Nurses’ perceptions on implementing a task-shifting/sharing strategy for hypertension management in patients with HIV in Nigeria: a group concept mapping study. Implement Sci Commu.

[43] Abdel-All M, Putica B, Praveen D, Abimbola S, Joshi R (2017). Effectiveness of community health worker training programmes for cardiovascular disease management in low-income and middle-income countries: a systematic review. BMJ Open.

[44] Rachlis B, Naanyu V, Wachira J, Genberg B, Koech B, Kamene R (2016). Community perceptions of community health workers (CHWs) and their roles in management for HIV, tuberculosis and hypertension in Western Kenya. PLoS ONE.

[45] Blackstone S, Iwelunmor J, Plange-Rhule J, Gyamfi J, Quakyi NK, Ntim M (2017). Sustaining nurse-led task-shifting strategies for hypertension control: a concept mapping study to inform evidence-based practice. Worldviews Evid Based Nur.

[46] Rawal LB, Kharel C, Yadav UN, Kanda K, Biswas T, Vandelanotte C (2020). Community health workers for non-communicable disease prevention and control in Nepal: a qualitative study. BMJ Open.

[47] Neupane D, Mclachlan CS, Gautam R, Mishra SR, Thorlund M, Schlütter M (2015). Literacy and motivation for the prevention and control of hypertension among female community health volunteers: a qualitative study from Nepal. Glob Health Action.

[48] Rawal LB, Kanda K, Biswas T, Tanim MI, Poudel P, Renzaho AM (2019). Non-communicable disease (NCD) corners in public sector health facilities in Bangladesh: a qualitative study assessing challenges and opportunities for improving NCD services at the primary healthcare level. BMJ Open.

[49] Long H, Huang W, Zheng P, Li J, Tao S, Tang S (2018). Barriers and facilitators of engaging community health workers in non-communicable disease (NCD) prevention and control in China: a systematic review (2006–2016). Int J Environ Res Public Health.

[50] Nebhinani M, Saini SK (2021). Leveraging role of non-physician health workers in prevention and control of non-communicable diseases in India: Enablers and challenges. J Family Med Prim Care.

[51] Joshi R, Alim M, Kengne AP, Jan S, Maulik PK, Peiris D (2014). Task shifting for non-communicable disease management in low and middle income countries–a systematic review. PLoS ONE.

[52] Odusola AO, Stronks K, Hendriks ME, Schultsz C, Akande T, Osibogun A (2016). Enablers and barriers for implementing high-quality hypertension care in a rural primary care setting in Nigeria: perspectives of primary care staff and health insurance managers. Glob Health Action.

[53] Teklehaimanot HD, Teklehaimanot A (2013). Human resource development for a community-based health extension program: a case study from Ethiopia. Hum Resour Health.

[54] Ramukumba MM (2020). Exploration of Community health workers’ views about in their role and support in primary health care in Northern Cape, South Africa. J Community Health.

